# Establishing a Quantitative Framework for Combined Oral Contraceptives: Evaluating the Impact of Drug–Drug Interactions on Exposure and Clinical (Surrogate) Efficacy Endpoints

**DOI:** 10.1002/jcph.70063

**Published:** 2025-07-22

**Authors:** Dain Chun, Huili Chen, Brian Cicali, Serge Guzy, Joachim Hoechel, Tianze Jiao, Valvanera Vozmediano, Stephan Schmidt

**Affiliations:** ^1^ Center for Pharmacometrics and Systems Pharmacology Department of Pharmaceutics College of Pharmacy University of Florida Orlando FL USA; ^2^ Pop‐Pharm Pharmacometrics Service Albany CA USA; ^3^ Bayer AG Berlin Germany; ^4^ Department of Pharmaceutical Outcomes and Policy College of Pharmacy University of Florida Gainesville FL USA; ^5^ Model Informed Development CTI Laboratories Covington KY USA

**Keywords:** combined oral contraceptives, drospirenone, drug–drug interactions, levonorgestrel, model‐based meta‐analysis, operational model of agonism, pharmacokinetics/pharmacodynamics, physiologically based pharmacokinetics

## Abstract

According to the FDA Guidance for Industry on Clinical Drug Interaction (DDI) Studies with Combined Oral Contraceptives (COCs), sponsors are expected to conduct dedicated clinical DDI studies if in vitro findings suggest weak or moderate CYP3A induction, while concomitant use of COCs with strong inducers should be avoided. The guidance further suggests that a negative DDI result for drospirenone (DRSP) may be extrapolated to other progestins that are less sensitive to CYP3A modulation, such as levonorgestrel (LNG). This approach assumes that DDI‐mediated changes in exposure directly translate into clinical efficacy across progestins. To evaluate the validity of this assumption, we established a quantitative link between dose, exposure, and response (Pearl Index [PI] and ovulation rate [OR]) via an integrated model‐based meta‐analysis, physiologically based pharmacokinetic, and pharmacokinetic/pharmacodynamic (PK/PD) modeling and simulation approach using data from 51 clinical studies in 36,040 women receiving LNG or DRSP. COCs containing LNG and DRSP were selected because they represent clinically relevant progestins at the lower and the upper end of the fraction metabolized via CYP3A4. The results of our analysis show a moderate correlation (Pearson's r = 0.52, 95% CI 0.46‐0.58, *P* < 0.001) between PI and OR, which enables the use of OR as an ethically measurable endpoint, even at subtherapeutic doses/exposures, to predict efficacy outcomes. They further show that DDI‐induced changes in exposure do not directly translate into clinical response. Therefore, DDIs with COCs should be interpreted in a PK/PD rather than a PK‐only context. The quantitative framework developed in this study can serve as the scientific basis to do so.

## Introduction

Drug–drug interactions (DDIs) with concomitant therapies can adversely impact the efficacy and safety of oral hormonal contraceptives (HCs) by affecting enzymes involved in their metabolism.^1^ DDIs with cytochrome P450 (CYP) 3A4 inducers are thought to be of particular importance in this regard because they decrease HC exposure to potentially inefficacious concentrations, thus resulting in an increased risk for unintended pregnancies.^1^, ^2^ The efficacy of HCs is routinely expressed as Pearl Index (PI), which also serves as the surrogate endpoint for pivotal clinical trials.^3^ PI is defined as the number of contraceptive failures per 100 woman‐years of HC treatment.^3^ However, quantifying the impact of CYP3A4 inducers on PI has been challenging because underlying dose–exposure–response relationships are not clinically established. This is because many HC drug products were developed decades ago, when the generation of such data was not required. Instead, HCs are dosed at the top of their respective exposure–response profiles and information on lower dose levels that could inform the impact of CYP3A4 inducers on PI is usually missing. The generation of such dose‐ranging PI data is also unethical because it would require exposing women to subtherapeutic HC concentrations that will result in unintended pregnancies amongst study participants. To overcome this limitation, our group previously established drug‐specific dose–PI relationships for two of the most used progestins, that is, levonorgestrel (LNG) and drospirenone (DRSP), using a model‐based meta‐analysis (MBMA) approach.^4^, ^5^ Once established, these dose–response relationships were translated into corresponding dose–exposure–response relationships using physiologically‐based pharmacokinetic (PBPK) models, which also allowed for the evaluation of DDIs on exposure and response.^4^, ^5^

Ovulation rate (OR), defined as the percentage of women who experience maturation of follicles and release eggs during a menstrual or estrous cycle, is a secondary efficacy endpoint used for dose finding during HC development.^6^, ^7^, ^8^, ^9^ In contrast to PI, dose–response is usually well understood for OR. However, to our knowledge, the relationship between OR and PI has not been readily established for any one progestin, let alone across progestins. To close this knowledge gap, we set out to link OR and PI across different progestins in a strictly quantitative fashion using the operational model of pharmacological agonism that Black and Leff^10^ first described in 1983. This model relies on a semi‐mechanistic description of the drug–receptor interaction as the basis for pharmacological response. It has been successfully used to rank‐order drugs of various drug classes according to their intrinsic efficacy.^11^ We now employ this concept in conjunction with an integrated MBMA and PBPK analysis approach to translate drug‐specific dose–exposure–response relationships into drug class‐specific dose–exposure–response relationships for OR and PI. Once established and verified, these relationships can be used to compare the impact of DDIs and BMI across progestins and to determine if differences in the extent of pharmacokinetic DDIs, which are due to differences in the fraction metabolized via CYP3A4, translate into clinically significant differences in PI between progestins.

## Methods

**Figure 1:**
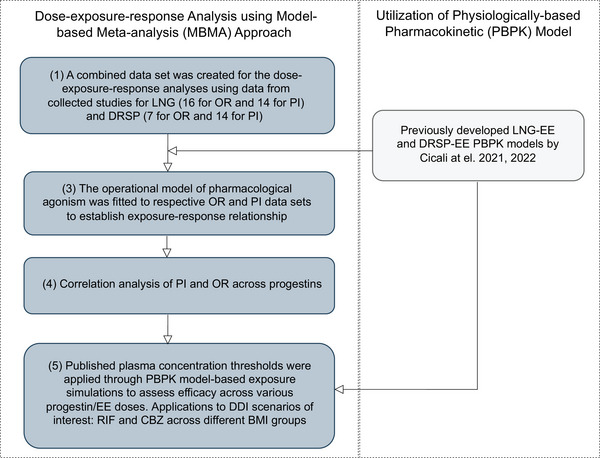
Model development workflow. (1) Clinical studies meeting inclusion criteria were identified and collected. (2) Dose endpoint datasets from these studies were used to simulate concentration datasets via previously developed physiologically‐based pharmacokinetic (PBPK) models by Cicali et al., incorporating progestin dosage levels and ethinyl estradiol (EE). (3) The operational pharmacological model of agonism was employed to establish exposure–response relationships. (4) Correlation analysis of PI and OR across progestins, LNG, and DRSP (5) The model was applied to evaluate drug–drug interaction (DDI) scenarios of interest, specifically with rifampicin (RIF) and carbamazepine (CBZ), across different body mass index (BMI) groups.

The study's objective was approached in a stepwise fashion, as outlined in Figure 1.

### Data

First, we conducted a systematic literature search for LNG and DRSP, including both combined oral contraceptives (COCs) and progestin‐only pills (POPs), to retrieve corresponding PI and OR data. These data were manually screened using inclusion and exclusion criteria outlined by Lingineni et al.,^12^ which considered factors such as the definition of endpoint measurements, participant age range, and study design. PI data was already available from previous analyses^12^ and was supplemented with respective OR data. OR was defined using follicle size and progesterone levels during the treatment cycle, including Hoogland score^6^ (Table S2). Detailed information on data collection as well as inclusion and exclusion criteria are provided in Figure S1.

### Establishing Exposure–Response Across Progestins for PI and OR

Second, we expanded the drug‐specific MBMA for LNG and PI by Lingineni et al.,^12^ which assumed only the progestin to be pharmacologically active, to a drug‐class‐specific (i.e., progestin‐specific) MBMA using the operational model of pharmacological agonism. Briefly, in the LNG‐specific MBMA by Lingineni et al.,^12^ (Equation 1),

(1) $$\begin{array}{ccc} {PI}_{\mathrm{ij}} & = & \mathrm{BL}\cdot \left(1-\;\frac{I_{\max}\;\cdot C_{\mathrm{avg},\mathrm{ij}}}{IC_{50,\mathrm{ij}}+C_{\mathrm{avg},\mathrm{ij}}}\right)+\varepsilon_{\mathrm{ij}},\\ & & \varepsilon_{\mathrm{ij}}\;∼\;N\left(0,\;\frac{\sigma_{i}^{2}}{n_{\mathrm{ij}}}\right) \end{array}$$

where ${PI}_{\mathrm{ij}}$ represents the PI of progestin type i (in this case, i is LNG) and trial and study arm j, BL represents the baseline PI in the absence of contraceptive use, $I_{\max}$ is the maximum fraction of suppression of the contraceptive efficacy, ${IC}_{50,\mathrm{ij}}$ is the average steady‐state concentration needed to achieve 50% maximum response, and $\varepsilon_{\mathrm{ij}}$ represents the additive residual error, which is assumed to follow a normal distribution with a mean of zero and variance ${\sigma_{i}}^{2}/n_{\mathrm{ij}}$, and $n_{\mathrm{ij}}$ denotes the number of patients receiving progestin i in trial and study arm j. Average steady‐state concentrations, C_avg,ij_, were obtained by translating dose into exposure via a previously developed PBPK model for LNG.^4^, ^5^

To expand this drug‐specific model for LNG into a drug‐class‐specific model for progestins (Equation 2), we accounted for differences in potency and the fact that different progestins share a downstream pathway upon receptor binding and activation.

Progestin‐specific receptor interactions were characterized by the dissociation constants ($K_{D,i}$), whereas the transducer function ($\tau_{i}$) serves as a practical measure of intrinsic efficacy.^13^ The higher the intrinsic efficacy, the more efficiently the drug produces a pharmacological response.^13^, ^14^ In Equation (2), $E_{\mathrm{ij}}$ represents the pharmacological response (PI or OR) for LNG or DRSP, in trial and in study arm j, BL represents the baseline pharmacological response in the absence of HC, $I_{\max}$ represents the maximum fractional decrease of pharmacological response from baseline, C_avg,ij_ represents the progestin‐specific average concentration, H the Hill factor, $K_{D,i}$ represents the dissociation constant for progestin i, $\varepsilon_{\mathrm{ij}}$ represents the additive residual error assuming a normal distribution with a mean of zero and variance ${\sigma_{i}}^{2}/n_{\mathrm{ij}}$, and $n_{\mathrm{ij}}$ denotes the number of patients receiving progestin i in trial and study arm j.

(2) $$\begin{array}{ccc} E_{\mathrm{ij}} & = & \mathrm{BL}\cdot \left(1-\;\frac{I_{\max}\;\cdot \;{\tau_{i}}^{H}\cdot \;{C_{\mathrm{avg},\mathrm{ij}}}^{H}}{{\left(K_{D,i}+C_{\mathrm{avg},\mathrm{ij}}\right)}^{H}+{\tau_{i}}^{n}\cdot \;{C_{\mathrm{avg},\mathrm{ij}}}^{H}}\right)\\ & & +\varepsilon_{\mathrm{ij}},\varepsilon_{\mathrm{ij}}\;∼\;N\left(0,\;\frac{\sigma_{i}^{2}}{n_{\mathrm{ij}}}\right) \end{array}$$

As a result, $K_{D,i}$ and C_avg,ij_ are drug‐specific parameters, whereas BL, $I_{\max}$, and H are systemic‐specific parameters. C_avg,ij_ is the average steady‐state concentration of LNG or DRSP that were derived through previously developed PBPK models.^4^, ^5^ To allow for a direct comparison of progestin effects at the receptor level, C_avg,ij_ data were converted into molar concentrations by accounting for progestin‐specific molecular weights.

The model was simultaneously fitted to PI data to estimate $\tau_{\mathrm{LNG}}$ and $\tau_{\mathrm{DRSP}}$, and the Hill factor H. The BL for PI was fixed to 85 and $I_{\max}$ was fixed to 1, assuming that all women in the study can become pregnant. $K_{D}$ values for LNG and DRSP were obtained from the literature.^15^ C_avg,ij_ and $K_{D,i}$ were log transformed (base 10) for this analysis. To account for the varying numbers of subjects in each arm across studies, PI and OR observations were weighted by the number of subjects in each study arm. Parameter estimation was performed using the maximum likelihood estimation method with a naïve pooled approach. The appropriateness of model fits was evaluated based on standard goodness‐of‐fit plots, precision, and physiological meaningfulness of parameter estimates. The workflow outlined above for PI was repeated for OR. Once the models were fitted to PI and OR separately, we evaluated if a correlation exists between the two progestins.

### Correlation Analysis Between PI and OR

To quantify the interplay between PI and OR, we simulated the variance–covariance matrix for the log‐transformed values of $\tau_{\mathrm{PI}}$ and $\tau_{\mathrm{OR}}$ for LNG and DRSP.

Let $Y_{1}$ be a random variable for $\tau_{\mathrm{PI}}$ and $Y_{2}$ be a random variable for $\tau_{\mathrm{OR}}$. Define 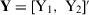 as a two‐component random vector sampled from a bivariate log‐normal distribution. Applying the logarithmic transformation, let $X_{i}=\log_{e}\left(Y_{i}\right)$, for i=1,2. The transformed variables $X_{i}$ follows a bivariate normal distribution with a mean vector 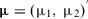 and variance–covariance matrix Σ, denoted as follows:

$$\;\left[\begin{array}{c} X_{1}\\ X_{2} \end{array}\right]=\left[\begin{array}{c} \log_{e}\left(Y_{1}\right)\\ \log_{e}\left(Y_{2}\right) \end{array}\right]\;\overset{\mathrm{iid}}{∼}N\left(\mu =\left[\begin{array}{c} \mu_{1}\\ \mu_{2} \end{array}\right],\Sigma =\left[\begin{array}{cc} \sigma_{11}^{2} & \sigma_{12}^{2}\\ \sigma_{21}^{2} & \sigma_{22}^{2} \end{array}\right]\right)$$

The probability density of Y is given by Equation (3):

(3)

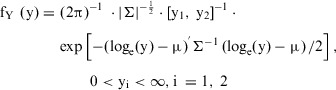

where 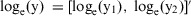 is a two‐component column vector.

Let $Y_{1}$ = 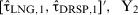
= 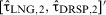, where these vectors represent estimates obtained from fitted models (Equation 2). These estimates are used to calculate the mean vector μ (Equation 4) and covariance matrix Σ (Equation 5) as follows:

(4) $$\mu =\;E\left(X\right)$$

(5)

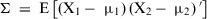




To this end, we created a 2 × 2 matrix (Equation 5), for which ($\tau_{\mathrm{PI}},\;\tau_{\mathrm{OR}}$) values for LNG and DRSP were obtained.

Given these assumptions, 500 $\tau_{\mathrm{PI}}$ and $\tau_{\mathrm{OR}}$ pairs for LNG and DRSP were simulated. A paired t‐test was used to compare respective means. Pearson's correlation coefficient was used to determine if a correlation exists between $\tau_{\mathrm{PI}}$ and $\;\tau_{\mathrm{OR}}$. The simulated pairs were utilized to fit models across progestins, integrating PI and OR. All analyses were performed in Phoenix version 8.4 (Certara, CA) and figures were generated in R (Version 4.3.3).

### Evaluating the Absolute and Relative Impact of CYP3A4 Inducers on PI and OR

Once developed and validated, the combined model was applied to assess the impact of the strong CYP3A4 index inducers, carbamazepine (CBZ) 400 mg and rifampicin (RIF) 600 mg,^1^ on PI and OR in women across three different BMI [kg/m^2^] groups: <25, 25‐30, and ≥30, for LNG and DRSP. Respective C_avg_ for LNG and DRSP in the presence and absence of these strong CYP3A4 index inducers were generated for 100 virtual subjects per BMI group via PBPK simulations,^4^, ^5^ which were then used as PK inputs into the combined model to predict the corresponding PI and OR values. The following doses were used for these simulations: LNG (100 or 150 µg in combination with either 20 or 30 µg of ethinyl estradiol [EE], respectively) and DRSP (3000 µg in combination with either 20 or 30 µg of EE). Incidence rate ratios (IRRs) were calculated for all evaluated DDI scenarios by dividing the incidence rate for each corresponding BMI group by that of the reference group (i.e., women with no CYP3A4 induction) and summarized graphically using a forest plot.

## Results

### Data

The final analysis dataset included information from 51 studies, a total of 36,040 women, and examined two different drugs across a wide dose range: LNG with 16 studies for OR (30, 90, 100, and 150 µg) and 14 studies for PI (90, 100, and 150 µg), and DRSP with 7 studies for OR (500, 1000, 2000, 3000, and 4000 µg), and 14 studies for PI (3000 and 4000 µg) up to a cutoff date of May 1, 2021. The dose–exposure–response data used in the analysis are available in Tables S2 and S3. Using the LNG‐EE and DRSP‐EE PBPK models, this translated to an average steady‐state concentration range of 0.54‐3.63 ng/mL for LNG (LNG 30 µg/EE0 to 150 µg/30 µg) and 5.47‐43.72 ng/mL for DRSP (500 to 4000 µg) (Tables S4 and S5).

### Establishing Exposure–Response Across Progestins for PI and OR

**Figure 2:**
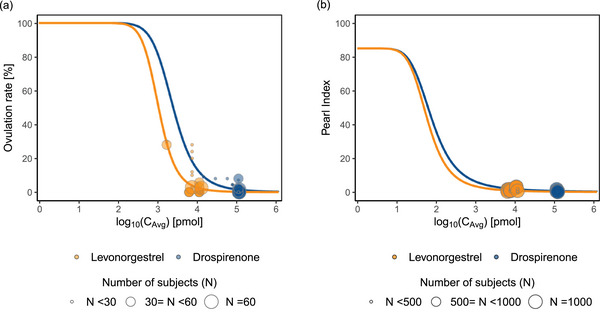
Exposure–response curves were fitted to the simulated concentration data. Observed (circles) and model‐predicted (line) of ovulation rate (OR) (a) and Pearl index (PI) (b) are shown. The number of subjects (N) in a study arm is represented by the size of the circle.

The operational model of agonism provided a good fit to the data, estimating corresponding parameter values, H, and operational factors of efficacy for LNG and DRSP, $\tau_{\mathrm{LNG}}$ and $\tau_{\mathrm{DRSP}}$ (Table 1, Figure 2).

**Table 1:** Parameter Estimates of Fitted Operational Model of Agonism model of Pearl Index (PI) and Ovulation Rate (OR)

	Pearl Index		Ovulation Rate	
Parameter	Estimate (CV%)	95% CI	Estimate (CV%)	95% CI
Hill factor (H)	9.653 (19.19)	(6.021, 13.285)	25.462 (1.69)	(24.616, 26.307)
$I_{\max}$	1 fixed	‐	1 fixed	‐
$\tau_{\mathrm{DRSP}}$	2.602 (9.59)	(2.113, 3.091)	1.867 (0.26)	(1.858, 1.878)
$\tau_{\mathrm{LNG}}$	3.046 (8.84)	(2.518, 3.574)	2.172 (0.19)	(2.164, 2.181)
$\log\left({K_{D}}_{\mathrm{DRSP}}\right)\;[\mathrm{mo}l^{-12}/L$]	2.949 fixed	‐	2.949 fixed	‐
$\log\left({K_{D}}_{\mathrm{LNG}}\right)[\mathrm{mo}l^{-12}/L$]	3.556 fixed	‐	3.556 fixed	‐
BL	85 fixed	‐	100 fixed	‐
$\sigma_{\mathrm{DRSP}}$	0.572 (2.57)	(0.543, 0.600)	2.260 (0.49)	(2.239, 2.282)
$\sigma_{\mathrm{LNG}}$	0.493 (2.56)	(0.468, 0.517)	6.572 (0.41)	(6.520, 6.625)

τ, transducer function; σ, additive residual error.

Model estimation yielded adequate results in terms of robustness, convergence, and parameter uncertainty. LNG showed a higher intrinsic efficacy for both PI ($\tau_{\mathrm{PI}}$) and OR ($\tau_{\mathrm{OR}}$) than DRSP, indicating that LNG more efficiently induces a pharmacological response than DRSP.

### Correlation Analysis Between PI and OR and Response Prediction

**Figure 3:**
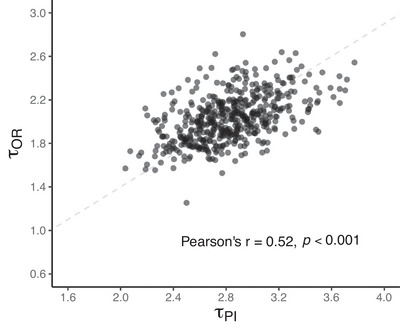
Correlation plot of simulated 500 pairs of operational factors of efficacy for ovulation rate (OR) and Pearl index (PI), $\;\tau_{\mathrm{OR}},$ and $\tau_{\mathrm{PI}}$. The plot displays the Pearson correlation coefficient (r) and the corresponding *P*‐value.

For simulated pairs, $\tau_{\mathrm{PI}}$ and $\;\tau_{\mathrm{OR}}$, the mean $\tau_{\mathrm{PI}}$ was 0.75 greater than the $\tau_{\mathrm{OR}}$ (95% CI 0.73‐0.77, paired t‐test *P* < 0.001). $\tau_{\mathrm{PI}}$ and $\tau_{\mathrm{OR}}$ had a moderate positive correlation (Pearson's r = 0.52, 95% CI 0.46‐0.58, *P* < 0.001) (Figure 3). For women with BMI < 25, the approved doses of LNG (100 µg/EE 20 µg and 150 µg/EE 30 µg) result in a simulated average PI of 2.61 to 3.25, whereas OR ranged from 28.5% to 34.3%, respectively. For DRSP (3000 µg /EE 20 µg and 3000 µg /EE 30 µg), we obtained simulated average PI of 0.55 and 0.56, whereas OR ranged from 2.65% to 2.74%, respectively (Table 2). For women in higher BMI groups (25 ≤ BMI < 30 and BMI ≥30), PI was estimated to be higher compared to women with BMI < 25. For women with 25 ≤ BMI < 30, the PI for LNG (100 µg/EE 20 µg and 150 µg/EE 30 µg) result in a simulated average 3.20 to 3.90, with OR from 33.8% to 40.6%. For DRSP at the same doses, the PI is 0.57 to 0.58, and the OR ranges from 2.77% to 2.87% (Table S3).

**Table 2:** Model‐Predicted Pearl Index (PI) for LNG and DRSP with Concomitant Use of CYP3A4 Inducers, CBZ 400 mg and RIF 600 mg, Across Various BMI Groups

				BMI [kg/m^2^]		
HC	DDI	Progestin Dose [µg]	EE Dose [µg]	< 25	25‐30	≥ 30
LNG	None (Ref)	100	20	3.25	3.90	4.00
		150	30	2.61	3.20	3.21
	CBZ	100	20	3.68 (13.2%)	4.57 (17.2%)	4.65 (16.3%)
		150	30	2.70 (3.4%)	3.27 (2.2%)	3.70 (15.3%)
	RIF	100	20	3.95 (21.5%)	4.92 (26.2%)	5.02 (25.5%)
		150	30	3.03 (16.1%)	3.74 (16.9%)	3.98 (24.0%)
DRSP	None (Ref)	3000	20	0.56	0.58	0.58
		3000	30	0.55	0.57	0.57
	CBZ	3000	20	0.68 (21.4%)	0.71 (22.4%)	0.73 (25.9%)
		3000	30	0.68 (23.6%)	0.70 (22.8%)	0.71 (24.6%)
	RIF	3000	20	0.73 (30.3%)	0.77 (32.8%)	0.79 (36.2%)
		3000	30	0.72 (30.9%)	0.75 (31.6%)	0.77 (35.1%)

CBZ, carbamazepine; DRSP, drospirenone; EE, ethinyl estradiol; LNG, levonorgestrel; RIF, rifampicin.

Results are presented as average predictions along with the percentage change indicating relative increase in efficacy with women with no CYP3A4 induction as reference.

### Evaluating the Relative and Absolute Impact of CYP3A4 Inducers on PI and OR

**Figure 4:**
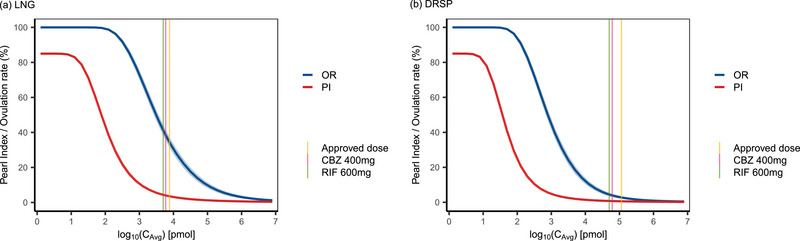
Exposure–response analysis of Pearl Index (PI) and ovulation rate (OR) for (a) LNG and (b) DRSP using operational model of agonism. The blue line represents the fitted model of OR and the red line represents the fitted model of PI, respectively. The vertical yellow line indicates progestin exposure when LNG100/EE20 (LNG 100 µg + EE 20 µg) or DRSP3000/EE20 (DRSP 3000 µg + EE 20 µg) without concurrent use of CYP3A4 inducers. The pink line indicates the progestin exposure when LNG100/EE20 or DRSP3000/EE20 is co‐administered with CBZ 400 mg, while the green line indicates the exposure when LNG100/EE20 or DRSP3000/EE20 is co‐administered with RIF 600 mg. CBZ, carbamazepine; DRSP, drospirenone; EE, ethinyl estradiol; LNG, levonorgestrel; RIF, rifampicin.

Our findings show that there was a higher relative change from baseline for PI in women with BMI < 25 for DRSP in the presence of strong CYP3A4 index inducers (CBZ: 1.21‐1.23 fold; RIF: 1.30 fold) compared to LNG (CBZ: 1.03‐1.13 fold; RIF: 1.16‐1.22 fold) as shown in Table 2 and Figure 4. This relationship was consistent in women with higher BMI (25 ≤ BMI < 30 and BMI ≥ 30), where the relative change from baseline for PI was higher for DRSP (25 ≤ BMI < 30, CBZ: 1.23 fold; RIF: 1.32‐1.33 fold; BMI > 30, CBZ: 1.24‐1.25 fold; RIF: 1.35 fold) compared to LNG (25 ≤ BMI <30, CBZ: 1.02‐1.17 fold; RIF: 1.17‐1.26 fold; BMI > 30, CBZ: 1.15‐1.16 fold; RIF: 1.24‐1.25 fold). However, when considering absolute values, DRSP is generally more efficacious than LNG, regardless of DDIs and BMI due to overall lower absolute PI values.

## Discussion

**Figure 5:**
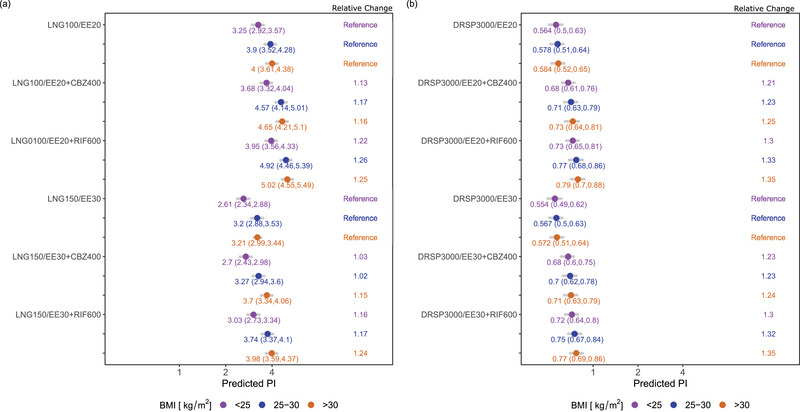
(a) Predicted Pearl Index (PI) with 95% CI of LNG150/EE30 (LNG 150 µg + EE 30 µg) and LNG100/EE20 (LNG 100 µg + EE 20 µg) co‐administered with CYP3A4 inducers across BMI groups. Reference group includes women with no CYP3A4 induction. (b) Predicted PI with 95% CI of DRSP3000/EE20 (DRSP 3000 µg + EE 20 µg) and DRSP3000/EE30 (DRSP 3000 µg + EE 30 µg) co‐administered with CYP3A4 inducers across BMI groups. Reference group includes women with no CYP3A4 induction. CBZ, carbamazepine; DRSP, drospirenone; EE, ethinyl estradiol; LNG, levonorgestrel; RIF, rifampicin.

According to the FDA Guidance for Industry on Clinical Drug Interaction Studies with Combined Oral Contraceptives, a sponsor ought to conduct a dedicated clinical DDI study if in vitro results suggest weak or moderate CYP3A induction potential for a new molecular entity, whereas labeling should recommend avoiding concomitant use with COCs in case of strong induction potential.^1^ The guidance further suggests that a negative DDI result for DRSP can be extrapolated to COCs containing progestins that are less sensitive to CYP3 induction (e.g., norethindrone or LNG) given that DRSP's metabolism is more sensitive to CYP3A modulation.^1^ However, these recommendations rely on the assumption that associated exposure changes translate directly into corresponding changes in response across progestins. The results of our analysis suggest that this assumption may be flawed and that a more differentiated evaluation may be needed. Although the results of our study suggest that the relative change in PI from baseline was more pronounced for DRSP than LNG in the presence of strong CYP3A4 index inducers in women with 25 ≤ BMI < 30, the PI baseline for DRSP is significantly lower than the PI baseline for LNG. In contrast, the absolute change in PI was more pronounced for LNG than for DRSP. This is because DRSP is dosed higher on its individual exposure–response profile than LNG (Figure 5), resulting in lower absolute PI values for DRSP, even in the presence of strong CYP3A4 inducers. These findings suggest that the impact of CYP3A induction on the efficacy of COCs should be interpreted in a PK/PD rather than a PK‐only context. Once real‐world evidence is taken into consideration, our previous analysis further suggests that the relative risk of contraception failure among patients with concomitant, strong CYP3A4‐inducing antiepileptics is in fact similar for LNG and DRSP, given that the respective rate ratio range (mean: 1.53, range: 0.94‐2.43) included one.^5^

The results of our analysis further suggest that CYP3A induction is not the only factor impacting progestin exposure. However, the interplay between the various factors, including CYP3A induction, BMI, EE dose, progestin exposure, and ultimately contraceptive efficacy, is complex and often not fully understood.^16^, ^17^, ^18^ This is because the underlying mechanisms are often nonlinear and, thus, difficult to extrapolate in the absence of a quantitative framework that integrates the various aspects in a strictly quantitative fashion. The establishment of such a framework requires the integration of data from different sources and the use of complementary analysis approaches. The use of the operational model of pharmacological agonism in conjunction with an integrated MBMA and PBPK analysis approach not only allowed us to establish dose–exposure–response across two different progestins but also to explore BMI and EE dose as PK sources of variability. It also allowed us to establish a link between the PI and OR using a variance–covariance analysis approach. The link between OR and PI effectively closes the knowledge gap for PI at subtherapeutic dose levels relevant to characterizing the impact of strong CYP3A inducers on the contraceptive efficacy of LNG and DRSP across a wide range of BMIs. It further allows us to link OR, that is, a more immediate and mechanistically relevant endpoint that is also ethically measurable at subtherapeutic doses/exposures to predicted efficacy outcomes, that is, PI.

Despite the progress made, we would also like to acknowledge some limitations of the current analysis to outline future analysis needs and objectives. First, there is considerable variability in the observed OR data for LNG (Table S2), which is partially due to the different methodologies used to detect ovulation. Closer evaluation of literature studies used in our analysis revealed that they strictly followed and recorded the established methodologies used to detect ovulation.^19^, ^20^ Nevertheless, a standardized definition of OR across clinical trials has the potential to further improve the precision and accuracy of using OR in contraceptive efficacy. Despite our best efforts to establish a consistent definition of the endpoint when conducting the analysis, there is still substantial residual variability, potentially introducing noise into the observations. While meta‐analysis is a powerful tool for combining the results of multiple, independent studies to produce a single, comprehensive analysis, one must account for the heterogeneity of study designs, populations, and study durations to avoid introducing bias into the analysis. Second, a direct comparison of PI values across studies is hindered by varying study durations. This is because a higher number of pregnancies is frequently observed at the early stages of the observation period, followed by a decrease in the number of pregnancies over time. This phenomenon has been attributed to different factors, including the more frequent sexual activity during initial study phases or the more consistent and correct use of contraception as the study continues. While PI remains the gold standard for evaluating contraceptive efficacy, it requires long‐term follow‐up and large sample sizes. In contrast, OR provides a more immediate and mechanistically relevant endpoint, as it reflects the ability of progestins to suppress ovulation via inhibition of the hypothalamic–pituitary–ovarian axis. This suppression is a primary mode of action for many COCs and supports the use of OR as an intermediate indicator of contraceptive efficacy. We acknowledge, however, that OR may not capture the full range of contraceptive mechanisms, such as thickening of cervical mucus, alterations in tubal motility, and changes in the endometrium, which may contribute to contraceptive efficacy but are not reflected by ovulation status. Third, different EE doses can contribute to the overall variability. We primarily considered them as a source of variability in PK in our analysis using integrated PBPK models. We acknowledge that EE may also contribute to contraceptive efficacy by thickening the endometrium. Dissecting the EE‐only effects from the COC effects is challenging because there are no EE‐only efficacy trials available. Finally, the current framework was informed using OR and PI data from LNG and DRSP and will have to be further verified with data from additional progestins, such as norethindrone and norgestimate. Our group is currently in the process of establishing respective PBPK and MBMA models for additional COCs to be included in the outlined framework. Inclusion of such data will allow for evaluating the broader applicability of the proposed framework across structurally and pharmacologically distinct progestins and contribute to a more comprehensive, mechanism‐based regulatory strategy for assessing the impact of DDIs on hormonal contraceptives.

In conclusion, the findings of our analysis suggest that PK‐only evaluation characterizing the impact of CYP3A induction on contraceptive efficacy paints a biased picture and should be replaced by a PK/PD‐based evaluation. The PK/PD framework established in the current analysis can help inform this assessment by serving as the scientific basis for integrating available information across progestins.

## Author Contributions

Dain Chun, Huili Chen, Brian Cicali, Serge Guzy, Joachim Hoechel, Tianze Jiao, Valvanera Vozmediano, and Stephan Schmidt wrote the manuscript. Dain Chun, Huili Chen, Brian Cicali, Serge Guzy, Joachim Hoechel, Tianze Jiao, Valvanera Vozmediano, and Stephan Schmidt designed the research. Dain Chun, Huili Chen, Brian Cicali, Serge Guzy, Joachim Hoechel, Tianze Jiao, Valvanera Vozmediano, and Stephan Schmidt performed the research. Dain Chun, Huili Chen, Brian Cicali, Serge Guzy, Joachim Hoechel, Tianze Jiao, Valvanera Vozmediano, and Stephan Schmidt analyzed the data.

## Conflicts of Interest

All other authors declared no competing interests for this work. IRB approval was not needed because the work is entirely based on published literature data. Patient consent was not needed because the work is entirely based on published literature data.

## Funding

This project was funded by grants (INV‐010213 and OPP1185454) provided by the Bill & Melinda Gates Foundation.

## Supporting information



Supporting Information

## Data Availability

N/A
