# Supplementary material for: Establishing a Quantitative Framework for Combined Oral Contraceptives: Evaluating the Impact of Drug–Drug Interactions on Exposure and Clinical (Surrogate) Efficacy Endpoints
Source: J Clin Pharmacol. 2025 Jul 22;65(12):1855–63. doi: 10.1002/jcph.70063 (PMC12649304; doi:10.1002/jcph.70063)
Supplement: Supplementary file 1 — Supporting Information [file JCPH-65-1855-s001.docx]

# **Supplementary Materials**

**Exploring Surrogate Endpoints in Oral Hormonal Contraceptive Efficacy Studies: Investigating the Correlation between Ovulation Rate and Pearl Index**

Dain Chun^1^, Huili Chen^1^, Brian Cicali^1^, Serge Guzy^2^, Joachim Hoechel^4^, Tianze Jiao^3^, Valvanera Vozmediano^1,5^, Stephan Schmidt^1^

^1^Center for Pharmacometrics and Systems Pharmacology, Department of Pharmaceutics, College of Pharmacy, University of Florida, Orlando, Florida, USA;

^2^Pop-Pharm Pharmacometrics Service, Albany, California, USA;

^3^Department of Pharmaceutical Outcomes and Policy, College of Pharmacy, University of Florida, Florida, USA;

^4^Bayer AG, Berlin, Germany;

^5^Model Informed Development, CTI Laboratories, Covington, KY 41011, USA

Corresponding author: Stephan Schmidt, sschmidt@cop.ufl.edu

6550 Sanger Road, Office 464

Orlando, FL, 32827, USA

Tel: (407) 313-7012

Keywords: Model-Based Meta-Analysis, Operational Model of Agonism, Levonorgestrel, Combined Oral Contraceptives, Drospirenone, Drug-Drug Interactions, Physiologically Based Pharmacokinetics, Pharmacokinetics/Pharmacodynamics

FUNDING

This project was funded by a grant (INV-010213 and OPP1185454) provided by the Bill & Melinda Gates Foundation.

# CONFLICTS OF INTEREST

All other authors declared no competing interests for this work.

IRB approval was not needed because the work is entirely based on published literature data.

Patient consent was not needed because the work is entirely based on published literature data.

Data sharing: N/A

**List of Tables**

**Table S1.** List of keywords used for study identification.

**Table S2.** Summary of studies included in the dose-exposure-response analyses for ovulation rate

**Table S3.** Summary of studies included in the dose-exposure-response analyses for Pearl Index

**Table S4.** Listing of observations of ovulation rate (OR) data set

**Table S5.** Listing of observations of Pearl Index (PI) data set

**Table S6.** Demographic characteristics of the simulated population usingphysiologically-based pharmacokinetic (PBPK) models.

**Table S7.** Model-predicted Pearl Index (PI) and ovulation rate (OR) for LNG and DRSP with concomitant use of CYP3A4 inducers, CBZ 400mg and RIF 600 mg, across various BMI groups.

**List of Figures**

**Figure S1.** Study Identification workflow for Model-Based Meta-Analysis (MBMA) data set creation.

**Figure S2.** Goodness-of-Fit plot of final operational model of agonism model for Pearl index (PI) and ovulation rate (OR) in exposure-response analysis.**Figure S3.** Predicted ovulation rate (OR) with 95% CI of LNG150/EE30 (LNG 150 µg + EE 30 µg) and LNG100/EE20 (LNG 100 µg + EE 20 µg) co-administered with CYP3A4 inducers across BMI groups.

**Table S1.** List of keywords used for study identification.

| The following keywords were entered to the querybox for LNG (05/01/2021):   1. “levonorgestrel” OR "Alesse" OR "Altavera" OR "Alysena" OR "Amethia" OR "Amethyst" OR "Ashlyna" OR "Aviane" OR "Camrese" OR "Chateal" OR "Climara Pro" OR "Cycle 21" OR "Daysee" OR "Emerres" OR "Enpresse" OR "Erlibelle" OR "Escapelle" OR "Falmina" OR "Introvale" OR "Isteranda" OR "Jadelle" OR "Jaydess" OR "Jolessa" OR "Klimonorm" OR "Kurvelo" OR "Kyleena" OR "Lessina" OR "Levlen" OR "Levodonna" OR "Levonelle" OR "Levonest" OR "Levosert" OR "Levora" OR "Liletta" OR "Loette" OR "Logynon" OR "LoSeasonique" OR "Lutera" OR "Lybrel" OR "Marlissa" OR "Microgynon" OR "Microlut" OR "Microvlar" OR "Min-Ovral" OR "Miranova" OR "Mirena" OR "My Way" OR "Myzilra" OR "Next Choice" OR "Nordette" OR "Norgeston" OR "NorLevo" OR "Norplant" OR "One Pill" OR "Option 2" OR "Orsythia" OR "Ovima" OR "Ovranette" OR "Plan B" OR "Plan B One-Step" OR "Portia" OR "Postinor" OR "Postinor-2" OR "Preventeza" OR "Ramonna" OR "Rigevidon" OR "Quartette" OR "Quasense" OR "Seasonale" OR "Seasonique" OR "Skyla" OR "Sronyx" OR "Tri-Levlen" OR "Trinordiol" OR "Triphasil" OR "Triquilar" OR "Tri-Regol" OR "Trivora" OR "Upostelle" OR "d-norgestrel" OR "d(–)-norgestrel" OR "D-norgestrel" OR "WY-5104" OR "SH-90999" 2. "ovulation" OR "inhibit ovulation" OR "ovulation inhibition" OR "inhibition of ovulation" OR "ovulation rate" OR "ovulate” 3. “pearl rate” OR “pearl index” OR “pregnancy rate” OR “pregnancy percentage” OR “number of pregnancies” OR “pregnancy” OR “pregnancy failure” OR “pregnant” 4. "clinical trials as topic"[MeSH Terms] OR "clinical trial"[Publication Type] OR "clinical trials as topic"[MeSH Terms] OR "observational study"[Publication Type] OR "observational studies as topic"[MeSH Terms] / **"Humans"[MeSH Terms]** 5. English[Language] 6. Review[Publication Type] OR letter[Publication Type] OR systematic review[Title/Abstract] 7. “emergency”   **#1 AND #2 AND #3 AND #4 AND #5 NOT #6 NOT #7** |
| --- |
| The following keywords were entered to the query box for DRSP (05/01/2021):   1. "Drospirenone" OR "dihydrospirorenone" OR "1,2-dihydro-spirorenone" OR "1,2-dihydrospirorenone" OR "ZK 30595" OR "ZK30595“ OR “Slynd” OR “Angeliq” OR “Yasmin” OR “Yasminelle” OR “Yaz” OR “Nextstellis” 2. "ovulation" OR "inhibit ovulation" OR "ovulation inhibition" OR "inhibition of ovulation" OR "ovulation rate" OR "ovulate” 3. “pearl rate” OR “pearl index” OR “pregnancy rate” OR “pregnancy percentage” OR “number of pregnancies” OR “pregnancy” OR “pregnancy failure” OR “pregnant” 4. "clinical trials as topic"[MeSH Terms] OR "clinical trial"[Publication Type] OR "clinical trials as topic"[MeSH Terms] OR "observational study"[Publication Type] OR "observational studies as topic"[MeSH Terms] / **"Humans"[MeSH Terms]** 5. English[Language] 6. Review[Publication Type] OR letter[Publication Type] OR systematic review[Title/Abstract] 7. “emergency”   **#1 AND #2 AND #3 AND #4 AND #5 NOT #6 NOT #7** |

**Table S2.** **Summary of studies included in the dose-exposure-response analyses for ovulation rate**

| Study ID | References | Year | Method of measurement | DOI |
| --- | --- | --- | --- | --- |
| 1 | Duijkers at el. | 2016 | Follicle and progesterone levels | DOI:10.1016/j.contraception.2015.12.007 |
| 2 | Duijkers at el. | 2015 | Follicular diameter, endometrial thickness, and serum estradiol (E2) and progesterone, Hoogland score | DOI:10.3109/13625187.2015.1044082 |
| 3 | Duijkers at el. | 2015 | Hoogland score | DOI:10.3109/13625187.2015.1074675 |
| 4 | Duijkers at el. | 2010 | Serum oestradiol, progesterone, follicle stimulating hormone (FSH) and luteinising hormone (LH), and ultrasonography of follicular diameter. | DOI:[10.3109/13625187.2010.504313](http://dx.doi.org/10.3109/13625187.2010.504313) |
| 5 | Seidman at el. | 2015 | Hoogland and Skouby (H/S) method. | DOI:10.1016/j.contraception.2015.03.001 |
| 6 | [Rosenbaum](https://www.tandfonline.com/author/Rosenbaum%2C+P) at el | 2000 | Hormonal parameters (LH, FSH, 17ß-estradiol and progesterone) and peripheral parameters (cervical score, spinnbarkeit and crystallization), as well as follicle size assessed by ultrasonography | [DOI:10.1080/13625180008500376](https://doi.org/10.1080/13625180008500376) |
| 7 | Klipping at el. | 2008 | Hoogland score | [DOI:10.1016/j.contraception.2008.02.019](https://doi.org/10.1016/j.contraception.2008.02.019) |
| 8 | Anzai at el. | 2011 | Hoogland score | [DOI:10.1016/j.contraception.2011.11.001](https://doi.org/10.1016/j.contraception.2011.11.001) |
| 9 | Barcellos at el. | 2019 | Serum progesterone levels | DOI:10.1016/j.contraception.2019.06.002 |
| 11 | [Csemiczky](https://pubmed.ncbi.nlm.nih.gov/?term=Csemiczky+G&cauthor_id=8863905) at el. | 1996 | Follicle-stimulating hormone and estradiol concentration and progesterone levels | DOI: 10.1007/BF01849631 |
| 12 | Young at el. | 2012 | Progesterone and estrogen levels | DOI:10.5414/CP201647 |
| 14 | Spona at el. | 1996 | Follicular size, the thickness of the endometrium as well as serum levels of LH, FSH, estradiol, and progesterone | DOI:10.1016/S0010-7824(96)00183-7 |
| 15 | Biswal at el. | 2013 | Follicle-stimulating hormone (FSH), luteinizing hormone (LH), estradiol, and progesterone concentrations | DOI:[10.5414/CP202142](https://doi.org/10.5414/cp202142) |
| 16 | Braun at el. | 2009 | Progesterone, oestradiol, luteinizing hormone, and follicle stimulating hormone serum concentrations | DOI:10.1111/j.1365-2125.2009.03468.x |
| 18 | [Cawello](https://onlinelibrary.wiley.com/authored-by/Cawello/Willi) at el. | 2013 | Progesterone serum concentration | DOI: 10.1111/epi.12085 |
| 20 | Archer at el. | 2009 | Serum 17beta-estradiol, progesterone, follicle-stimulating hormone and luteinizing hormone concentrations | DOI: 10.1016/j.contraception.2009.03.006 |
| 22 | [Davis](https://pubmed.ncbi.nlm.nih.gov/?term=Davis%20AR%5BAuthor%5D) at el. | 2011 | ovarian follicular diameters, vaginal ultrasounds, and progesterone levels | DOI: 10.1111/j.1528-1167.2010.02917.x |
| 24 | Rice at el. | 1999 | Transvaginal ultrasonography and serum oestradiol, progesterone, luteinizing hormone (LH) and follicle stimulating hormone (FSH) | DOI:10.1093/humrep/14.4.982 |
| 26 | Pierson at el. | 2003 | Follicular size | DOI:10.1016/s0015-0282(03)00556-9 |
| 27 | Kroll at el. | 2014 | Hoogland and Skouby method | DOI:10.3109/13625187.2014.979282 |
| 30 | Olsson at el. | 2001 | Serum levels of estradiol and progesterone | DOI:10.1016/S0149-2918(00)89083-9 |
| 31 | Westhoff at el. | 2010 | Follicular development and endogenous E2 levels | DOI:10.1097/AOG.0b013e3181e79440 |
| 33 | Lakha at el. | 2007 | Progesterone level | DOI:10.1093/humrep/dem177 |
| 34 | Duijkers at el. | 2004 | Follicular diameter and serum hormone concentrations (FSH, LH, 17β-estradiol, progesterone) | DOI:10.1093/humrep/deh494 |
| 38 | Coney at el. | 1999 | Serum progesterone level | DOI:10.1016/S0002-9378(99)70364-9 |

**Table S3.** **Summary of studies included in the dose-exposure-response analyses for Pearl Index**

| Study ID | References | Year | DOI |
| --- | --- | --- | --- |
| 326 | Westhoff at el. | 2012 | DOI: 10.1097/AOG.0b013e318250c3a0 |
| 331 | [Klipping](https://pubmed.ncbi.nlm.nih.gov/?term=Klipping+C&cauthor_id=22454003) at el. | 2012 | DOI: 10.1136/jfprhc-2011-100213 |
| 339 | Jensen at el. | 2012 | DOI: 10.1016/j.contraception.2011.12.009 |
| 354 | [Mansour](https://www.tandfonline.com/author/Mansour%2C+Diana) at el. | 2011 | [DOI: 10.3109/13625187.2011.614029](https://doi.org/10.3109/13625187.2011.614029) |
| 426 | Guang-Sheng at el. | 2010 | DOI: [10.1007/BF03256908](https://doi.org/10.1007/bf03256908) |
| 584 | Cibula at el. | 2006 | DOI: [10.2165/00044011-200626030-00004](https://doi.org/10.2165/00044011-200626030-00004) |
| 2125 | Wiegratz at el. | 2015 | DOI: [10.2147/IJWH.S71906](https://doi.org/10.2147/ijwh.s71906) |
| 2479 | Westhoff at el. | 2011 | DOI: 10.1016/j.fertnstert.2011.07.148 |
| 2618 | Hernádi at el. | 2009 | [DOI: 10.1016/j.contraception.2009.01.016](https://doi.org/10.1016/j.contraception.2009.01.016) |
| 123 | Archer at el. | 2015 | [DOI: 10.1016/j.contraception.2015.07.014](https://doi.org/10.1016/j.contraception.2015.07.014) |
| 3197 | Parsey at el. | 2000 | [DOI: 10.1016/S0010-7824(00)00083-4](https://doi.org/10.1016/S0010-7824(00)00083-4) |
| 440 | Anttila at el. | 2009 | [DOI: 10.1016/j.contraception.2009.03.013](https://doi.org/10.1016/j.contraception.2009.03.013) |
| 59 | [Borgatta](https://www.tandfonline.com/author/Borgatta%2C+Lynn) at el. | 2016 | [DOI:10.1080/13625187.2016.1212987](https://doi.org/10.1080/13625187.2016.1212987) |
| 964 | [J. Huber](https://www.tandfonline.com/author/Huber%2C+J) at el. | 2000 | [DOI:10.1080/13625180008500375](https://doi.org/10.1080/13625180008500375) |
| 4670 | A Teichmann at el. | 2009 | DOI: 10.1016/j.contraception.2009.05.128 |
| 591 | DF Archer at el. | 2009 | [DOI:10.1016/j.contraception.2006.07.005](https://doi.org/10.1016/j.contraception.2006.07.005) |
| 435 | R Kroll at el. | 2010 | DOI: [10.1016/j.contraception.2009.07.003](https://doi.org/10.1016/j.contraception.2009.07.003) |
| 1036 | PJ Boerrigter | 1999 | DOI: [10.1016/s0149-2918(00)88272-7](https://doi.org/10.1016/s0149-2918(00)88272-7) |
| 1087 | [R Bannemerschult](https://pubmed.ncbi.nlm.nih.gov/?term=Bannemerschult+R&cauthor_id=9437556) at el. | 1997 | DOI: [10.1016/s0010-7824(97)00157-1](https://doi.org/10.1016/s0010-7824(97)00157-1) |
| 1103 | [D F Archer](https://pubmed.ncbi.nlm.nih.gov/?term=Archer+DF&cauthor_id=9115001) at el. | 1997 | DOI: [10.1016/s0010-7824(97)00024-3](https://doi.org/10.1016/s0010-7824(97)00024-3) |
| 1000 | [D F Archer](https://pubmed.ncbi.nlm.nih.gov/?term=Archer+DF&cauthor_id=9115001) at el. | 1999 | DOI: [10.1016/s0002-9378(99)70362-5](https://doi.org/10.1016/s0002-9378(99)70362-5) |
| 4297 | MJ Rosenberg at el. | 1999 | DOI: [10.1016/s0010-7824(99)00109-2](https://doi.org/10.1016/s0010-7824(99)00109-2) |
| 4346 | [J Endrikat](https://pubmed.ncbi.nlm.nih.gov/?term=Endrikat+J&cauthor_id=11535206) at el. | 2001 | DOI: [10.1016/s0010-7824(01)00221-9](https://doi.org/10.1016/s0010-7824(01)00221-9) |
| 4670 | A Teichmann at el. | 2009 | DOI: [10.1016/j.contraception.2009.05.128](https://doi.org/10.1016/j.contraception.2009.05.128) |
| 641 | FD Anderson at el. | 2006 | DOI: [10.1016/j.contraception.2005.09.010](https://doi.org/10.1016/j.contraception.2005.09.010) |
| 4451 | FD Anderson at el. | 2003 | DOI: [10.1016/s0010-7824(03)00141-0](https://doi.org/10.1016/s0010-7824(03)00141-0) |

**Table S4.** Listing of observations of ovulation rate (OR) data set

| ID | Study ID | Study Arm | Mean Age  [year] | BMI  [kg/m^2^] | N of Subjects | Progestin Type | Progestin Dose  [µg] | C_avg_ [ng/mL] | EE Dose  [µg] | Study Length [day] | Outcome [%] |
| --- | --- | --- | --- | --- | --- | --- | --- | --- | --- | --- | --- |
| 1 | 1 | 1 | 27.9 | 23.19 | 62 | DRSP | 4000 | 43.72 | 0 | 28 | 0 |
| 2 | 1 | 1 | 27.9 | 23.19 | 60 | DRSP | 4000 | 43.72 | 0 | 56 | 1.6 |
| 3 | 1 | 2 | 28.2 | 23.91 | 65 | DRSP | 4000 | 43.72 | 0 | 28 | 0 |
| 4 | 1 | 2 | 28.2 | 23.91 | 63 | DRSP | 4000 | 43.72 | 0 | 56 | 0 |
| 5 | 2 | 1 | 27.2 | 24.11 | 27 | DRSP | 4000 | 43.72 | 0 | 28 | 0 |
| 6 | 2 | 1 | 27.2 | 24.11 | 27 | DRSP | 4000 | 43.72 | 0 | 56 | 3.7 |
| 7 | 3 | 1 | 24.3 | 22.54 | 17 | DRSP | 3000 | 36.58 | 5 | 28 | 0 |
| 8 | 3 | 1 | 24.3 | 22.54 | 17 | DRSP | 3000 | 36.58 | 5 | 84 | 0 |
| 9 | 3 | 2 | 23.7 | 23.2 | 19 | DRSP | 3000 | 38.79 | 10 | 28 | 0 |
| 10 | 3 | 2 | 23.7 | 23.2 | 19 | DRSP | 3000 | 38.79 | 10 | 84 | 0 |
| 11 | 3 | 3 | 23.4 | 23.03 | 20 | DRSP | 3000 | 41.97 | 20 | 28 | 0 |
| 12 | 3 | 3 | 23.4 | 23.03 | 20 | DRSP | 3000 | 41.97 | 20 | 84 | 0 |
| 13 | 4 | 2 | 22.9 | 22.3 | 16 | DRSP | 3000 | 44.43 | 30 | 28 | 0 |
| 14 | 4 | 2 | 22.9 | 22.3 | 16 | DRSP | 3000 | 44.43 | 30 | 56 | 0 |
| 15 | 4 | 2 | 22.9 | 22.3 | 15 | DRSP | 3000 | 44.43 | 30 | 168 | 0 |
| 16 | 5 | 2 | 26.4 | 24 | 48 | DRSP | 3000 | 41.97 | 20 | 28 | 0 |
| 17 | 5 | 2 | 26.4 | 24 | 45 | DRSP | 3000 | 41.97 | 20 | 56 | 0 |
| 18 | 5 | 2 | 26.4 | 24 | 43 | DRSP | 3000 | 41.97 | 20 | 84 | 0 |
| 19 | 6 | 1 | -- | -- | 11 | DRSP | 500 | 5.47 | 0 | 28 | 9 |
| 20 | 6 | 2 | -- | -- | 12 | DRSP | 1000 | 10.93 | 0 | 56 | 8 |
| 21 | 6 | 3 | -- | -- | 12 | DRSP | 2000 | 21.86 | 0 | 84 | 8 |
| 22 | 6 | 4 | -- | -- | 11 | DRSP | 3000 | 32.79 | 0 | 28 | 0 |
| 23 | 6 | 5 | -- | -- | 23 | DRSP | 2000 | 29.62 | 30 | 28 | 4.4 |
| 24 | 6 | 5 | -- | -- | 23 | DRSP | 2000 | 29.62 | 30 | 56 | 4.4 |
| 25 | 6 | 5 | -- | -- | 23 | DRSP | 2000 | 29.62 | 30 | 84 | 4.4 |
| 26 | 6 | 6 | -- | -- | 23 | DRSP | 3000 | 44.43 | 30 | 28 | 0 |
| 27 | 6 | 6 | -- | -- | 23 | DRSP | 3000 | 44.43 | 30 | 56 | 0 |
| 28 | 6 | 6 | -- | - | 23 | DRSP | 3000 | 44.43 | 30 | 84 | 0 |
| 29 | 7 | 1 | 25.6 | 22.2 | 50 | DRSP | 3000 | 41.97 | 20 | 56 | 0 |
| 30 | 7 | 1 | 25.6 | 22.2 | 50 | DRSP | 3000 | 41.97 | 20 | 84 | 2 |
| 31 | 7 | 2 | 25.6 | 23 | 50 | DRSP | 3000 | 41.97 | 20 | 56 | 2 |
| 32 | 7 | 2 | 25.6 | 23 | 50 | DRSP | 3000 | 41.97 | 20 | 84 | 8 |
| 33 | 8 | 1 | 27.9 | 21.2 | 18 | DRSP | 3000 | 41.97 | 20 | 28 | 0 |
| 34 | 8 | 1 | 27.9 | 21.2 | 18 | DRSP | 3000 | 41.97 | 20 | 56 | 0 |
| 35 | 8 | 2 | 23.8 | 22.5 | 23 | DRSP | 3000 | 41.97 | 20 | 28 | 0 |
| 36 | 8 | 2 | 23.8 | 22.5 | 23 | DRSP | 3000 | 41.97 | 20 | 56 | 7.1 |
| 37 | 11 | 1 | 28.2 | 22.1 | 10 | LNG | 150 | 3.63 | 30 | 28 | 0 |
| 38 | 11 | 2 | 28.2 | 22.1 | 10 | LNG | 150 | 3.63 | 30 | 28 | 0 |
| 39 | 12 | 1 | 28.5 | 23.3 | 15 | LNG | 150 | 3.63 | 30 | 28 | 0 |
| 40 | 12 | 2 | 28.5 | 23.3 | 15 | LNG | 150 | 3.63 | 30 | 28 | 0 |
| 41 | 14 | 1 | 27.5 | -- | 24 | LNG | 150 | 3.53 | 20 | 84 | 0 |
| 42 | 15 | 1 | 22.3 | 22.6 | 23 | LNG | 150 | 3.63 | 30 | 28 | 0 |
| 43 | 15 | 1 | 22.3 | 22.6 | 23 | LNG | 150 | 3.63 | 30 | 56 | 0 |
| 44 | 16 | 1 | 27.1 | 23.6 | 21 | LNG | 150 | 3.63 | 30 | 28 | 0 |
| 45 | 16 | 2 | 27.1 | 23.6 | 22 | LNG | 150 | 3.63 | 30 | 28 | 0 |
| 46 | 18 | 1 | 30.5 | 22.12 | 31 | LNG | 150 | 3.63 | 30 | 28 | 0 |
| 47 | 18 | 1 | 30.5 | 22.12 | 31 | LNG | 150 | 3.63 | 30 | 56 | 0 |
| 48 | 20 | 1 | 27.07 | 25.75 | 58 | LNG | 90 | 2.02 | 20 | 28 | 0 |
| 49 | 20 | 1 | 27.07 | 25.75 | 58 | LNG | 90 | 2.02 | 20 | 56 | 0 |
| 50 | 20 | 1 | 27.07 | 25.75 | 58 | LNG | 90 | 2.02 | 20 | 84 | 0 |
| 51 | 22 | 2 | 24.7 | 23.9 | 10 | LNG | 100 | 2.39 | 20 | 56 | 10 |
| 52 | 24 | 2 | 27.6 | -- | 57 | LNG | 30 | 0.54 | 0 | 336 | 28 |
| 53 | 26 | 3 | 25.6 | -- | 25 | LNG | 100 | 2.39 | 20 | 28 | 12 |
| 54 | 26 | 3 | 25.6 | -- | 25 | LNG | 100 | 2.39 | 20 | 56 | 24 |
| 55 | 26 | 3 | 25.6 | -- | 25 | LNG | 100 | 2.39 | 20 | 84 | 28 |
| 56 | 26 | 3 | 25.6 | -- | 25 | LNG | 100 | 2.39 | 20 | 140 | 20 |
| 57 | 27 | 1 | 27.6 | 23.39 | 35 | LNG | 150 | 3.63 | 30 | 91 | 0 |
| 58 | 27 | 1 | 27.6 | 23.39 | 35 | LNG | 150 | 3.63 | 30 | 91 | 2.9 |
| 59 | 27 | 1 | 27.6 | 23.39 | 35 | LNG | 150 | 3.63 | 30 | 91 | 5.7 |
| 60 | 30 | 1 | 30 | -- | 12 | LNG | 150 | 3.63 | 30 | 28 | 0 |
| 61 | 30 | 2 | 30 | -- | 12 | LNG | 150 | 3.63 | 30 | 28 | 0 |
| 62 | 31 | 2 | 25.3 | 28.25 | 70 | LNG | 100 | 2.39 | 20 | 112 | 2.9 |
| 63 | 31 | 2 | 25.1 | 28.15 | 80 | LNG | 150 | 3.63 | 30 | 112 | 2.5 |
| 64 | 33 | 1 | 30.4 | 22.4 | 23 | LNG | 30 | 0.54 | 0 | 168 | 30 |
| 65 | 34 | 1 | 28 | 23.2 | 19 | LNG | 150 | 3.63 | 30 | 84 | 0 |
| 66 | 38 | 1 | 29 | -- | 26 | LNG | 100 | 2.39 | 20 | 112 | 2.7 |

**Table S5.** Listing of observations of Pearl Index (PI) data set

| ID | Study ID | Study Arm | Mean Age  [year] | BMI  [kg/m^2^] | N of Subjects | Progestin Type | Progestin Dose [µg] | C_avg_  [ng/mL] | EE Dose  [µg] | Study Length  [day] | Outcome  [%] |
| --- | --- | --- | --- | --- | --- | --- | --- | --- | --- | --- | --- |
| 1 | 326 | 2 | 27.8 | 24.5 | 545 | DRSP | 3000 | 44.43 | 30 | 364 | 1.83 |
| 2 | 326 | 2 | -- | -- | 463 | DRSP | 3000 | 44.43 | 30 | 364 | 1.89 |
| 3 | 331 | 1 | 24.8 | 22.5 | 626 | DRSP | 3000 | 41.97 | 20 | 720 | 0.64 |
| 4 | 339 | 1 | 25.2 | 24.1 | 1406 | DRSP | 3000 | 41.97 | 20 | 360 | 1.65 |
| 5 | 354 | 2 | 28 | 23 | 534 | DRSP | 3000 | 44.43 | 30 | 364 | 0.66 |
| 6 | 354 | 2 | -- | -- | 442 | DRSP | 3000 | 44.43 | 30 | 364 | 0.81 |
| 7 | 426 | 1 | 30 | 21.5 | 573 | DRSP | 3000 | 44.43 | 30 | 364 | 0.584 |
| 8 | 426 | 1 | 30 | 21.5 | 573 | DRSP | 3000 | 44.43 | 30 | 364 | 0.208 |
| 9 | 584 | 1 | 24.6 | 22.4 | 516 | DRSP | 3000 | 41.97 | 20 | 729 | 0.23 |
| 10 | 584 | 1 | 24.6 | 22.4 | 516 | DRSP | 3000 | 41.97 | 20 | 729 | 0.12 |
| 11 | 2125 | 1 | 25.3 | 22.7 | 250 | DRSP | 3000 | 41.97 | 20 | 360 | 0 |
| 12 | 2125 | 2 | 25.7 | 22.4 | 249 | DRSP | 3000 | 41.97 | 20 | 360 | 0 |
| 13 | 2479 | 2 | -- | -- | 554 | DRSP | 3000 | 44.43 | 30 | 364 | 1.89 |
| 14 | 2618 | 1 | 24.7 | 21.7 | 977 | DRSP | 3000 | 41.97 | 20 | 364 | 0.22 |
| 15 | 2618 | 1 | 24.7 | 21.7 | 1101 | DRSP | 3000 | 41.97 | 20 | 364 | 0.49 |
| 16 | 2618 | 1 | -- | -- | 700 | DRSP | 3000 | 41.97 | 20 | 364 | 0.81 |
| 17 | 2618 | 1 | -- | -- | 401 | DRSP | 3000 | 41.97 | 20 | 364 | 0.31 |
| 18 | 123 | 1 | 28.7 | 23 | 713 | DRSP | 4000 | 43.72 | 0 | 364 | 0.5423 |
| 19 | 123 | 1 | 28.7 | 23 | 713 | DRSP | 4000 | 43.72 | 0 | 364 | 0.5106 |
| 20 | 123 | 1 | -- | -- | 569 | DRSP | 4000 | 43.72 | 0 | 364 | 0.7052 |
| 21 | 3197 | 1 | 26.4 | -- | 220 | DRSP | 3000 | 44.43 | 30 | 364 | 0.455 |
| 22 | 3197 | 1 | 26.4 | -- | 326 | DRSP | 3000 | 44.43 | 30 | 364 | 0.406 |
| 23 | 3197 | 1 | 26.4 | -- | 326 | DRSP | 3000 | 44.43 | 30 | 364 | 0.407 |
| 24 | 440 | 1 | 25.2 | 22.3 | 229 | DRSP | 3000 | 41.97 | 20 | 196 | 0 |
| 25 | 59 | 2 | 23.9 | 23.9 | 281 | DRSP | 3000 | 44.43 | 30 | 540 | 0.91 |
| 26 | 59 | 2 | 23.9 | 23.9 | 281 | DRSP | 3000 | 44.43 | 30 | 540 | 1.82 |
| 27 | 964 | 1 | 25.2 | -- | 1657 | DRSP | 3000 | 44.43 | 30 | 364 | 0.71 |
| 28 | 964 | 1 | 25.2 | -- | 1657 | DRSP | 3000 | 44.43 | 30 | 364 | 0.07 |
| 29 | 4670 | 1 | 27.6 | 23 | 323 | LNG | 90 | 2.02 | 20 | 360 | 0 |
| 30 | 591 | 1 | 28.8 | 26 | 2134 | LNG | 90 | 2.02 | 20 | 360 | 1.6 |
| 31 | 591 | 1 | 28.8 | 26 | 2134 | LNG | 90 | 2.02 | 20 | 360 | 1.26 |
| 32 | 591 | 1 | 28.8 | 26 | 2134 | LNG | 90 | 2.02 | 20 | 360 | 0.34 |
| 33 | 435 | 1 | 27.7 | 27 | 1724 | LNG | 100 | 2.28 | 20/10 | 364 | 1.73 |
| 34 | 1036 | 1 | 26 | -- | 1560 | LNG | 100 | 2.39 | 20 | 168 | 0.65 |
| 35 | 1087 | 1 | 25.6 | -- | 805 | LNG | 100 | 2.39 | 20 | 168 | 0.29 |
| 36 | 1103 | 1 | 27 | -- | 1477 | LNG | 100 | 2.39 | 20 | 360 | 0.84 |
| 37 | 1000 | 1 | 27.2 | -- | 1708 | LNG | 100 | 2.39 | 20 | 1095 | 0.88 |
| 38 | 4297 | 1 |  | -- | 154 | LNG | 100 | 2.39 | 20 | 168 | 1.5 |
| 39 | 4346 | 1 | 25.3 | -- | 380 | LNG | 100 | 2.39 | 20 | 364 | 0.9 |
| 40 | 4670 | 2 | 27.2 | 23 | 318 | LNG | 100 | 2.39 | 20 | 364 | 1.19 |
| 42 | 641 | 1 | -- | -- | 1006 | LNG | 150 | 3.63 | 30/10 | 364 | 0.78 |
| 43 | 4346 | 3 | 26.1 | -- | 125 | LNG | 150 | 3.63 | 30 | 364 | 0 |
| 44 | 4451 | 1 | 27.8 | -- | 456 | LNG | 150 | 3.63 | 30 | 364 | 0.6 |
| 45 | 4451 | 2 | 27.83 | 26 | 226 | LNG | 150 | 3.63 | 30 | 364 | 1.78 |

**Table S6.** Demographic information on simulated population using physiologically based pharmacokinetic (PBPK) models.

|  | Healthy Population | Overweight Population | Obese Population |
| --- | --- | --- | --- |
| Weight [kg] | 44 – 76 | 50 – 94 | 65 – 108 |
| BMI [kg/m^2^] | 18.5 – 24.5 | 25 – 29.8 | 30.1 – 41.5 |
| Age [year] | 18 – 40 | 18 – 40 | 18 – 40 |
| Height [cm] | 145 – 182 | 135 – 185 | 141 – 176 |

**Table S7.** Model-predicted Pearl Index and ovulation rate for LNG and DRSP with concomitant use of CYP3A4 inducers, CBZ 400mg and RIF 600 mg, across various BMI groups. Results are presented as average predictions along with 95% CI assuming women with no CYP3A4 induction as reference**.**

| HCA | DDI | Progestin Dose [µg] | EE Dose [µg] | BMI < 25 | | 25 ≤ BMI < 30 | | BMI ≥ 30 | |
| --- | --- | --- | --- | --- | --- | --- | --- | --- | --- |
|  |  |  |  | PI (95% CI) | OR (95% CI) | PI (95% CI) | OR (95% CI) | PI (95% CI) | OR (95% CI) |
| LNG | None | 100 | 20 | 3.25  (2.92,3.57) | 34.33  (31.36,37.29) | 3.90  (3.52,4.28) | 40.59 (37.50,43.68) | 4.00  (3.61,4.38) | 41.02  (37.92,44.12) |
|  |  | 150 | 30 | 2.61  (2.34,2.88) | 28.45  (25.67,31.23) | 3.20  (2.88,3.53) | 33.77  (31.69,35.86) | 3.21  (2.99,3.44) | 34.08  (31.12,37.04) |
|  | CBZ | 100 | 20 | 3.68  (3.32,4.04) | 38.39  (35.34,41.45) | 4.57  (4.14,5.01) | 45.78  (42.63,48.92) | 4.65  (4.21,5.10) | 46.41  (43.27,49.56) |
|  |  | 150 | 30 | 2.70  (2.43,2.98) | 31.95  (29.05,34.85) | 3.27 (2.94,3.60) | 38.20  (35.15,41.26) | 3.70  (3.34,4.06) | 38.73  (35.67,41.79) |
|  | RIF | 100 | 20 | 3.95  (3.56,4.33) | 40.90  (37.81,44.00) | 4.92  (4.46,5.39) | 48.45  (45.30 51.60) | 5.02  (4.55,5.49) | 49.15  (46.00,52.30) |
|  |  | 150 | 30 | 3.03 (2.73,3.34) | 33.67  (30.72,36.62) | 3.74  (3.37,4.10) | 40.20  (37.11,43.28) | 3.98 (3.59,4.37) | 40.90  (37.81,44.00) |
| DRSP | None | 3000 | 20 | 0.56  (0.50,0.63) | 2.74  (1.98,3.50) | 0.58 (0.51,0.64) | 2.87  (2.09,3.65) | 0.58 (0.52,0.65) | 2.92  (2.13,3.71) |
|  |  | 3000 | 30 | 0.55  (0.49,0.62) | 2.65  (1.90,3.40) | 0.57  (0.50,0.63) | 2.77 (2.00,3.54) | 0.57 (0.51,0.64) | 2.82  (2.04,3.59) |
|  | CBZ | 3000 | 20 | 0.68  (0.61,0.76) | 3.92  (2.98,4.85) | 0.71 (0.63,0.79) | 4.19  (3.22,5.16) | 0.73 (0.64,0.81) | 4.35  (3.36, 5.35) |
|  |  | 3000 | 30 | 0.68  (0.60,0.75) | 3.82  (2.90,4.75) | 0.70 (0.62,0.78) | 4.08  (3.12,5.03) | 0.71  (0.63,0.79) | 4.22  (3.24,5.19) |
|  | RIF | 3000 | 20 | 0.73  (0.65,0.81) | 4.43  (3.42,5.43) | 0.77  (0.68,0.86) | 4.83  (3.78,5.89) | 0.79 (0.70,0.88) | 4.89  (3.82,5.95) |
|  |  | 3000 | 30 | 0.72  (0.64,0.80) | 4.28  (3.29,5.26) | 0.75  (0.67,0.84) | 4.65  (3.62, 5.69) | 0.77 (0.69,0.86) | 5.09  (4.00,6.17) |

CBZ, carbamazepine; RIF, rifampicin; EE, ethinyl estradiol; LNG, levonorgestrel; DRSP, drospirenone.


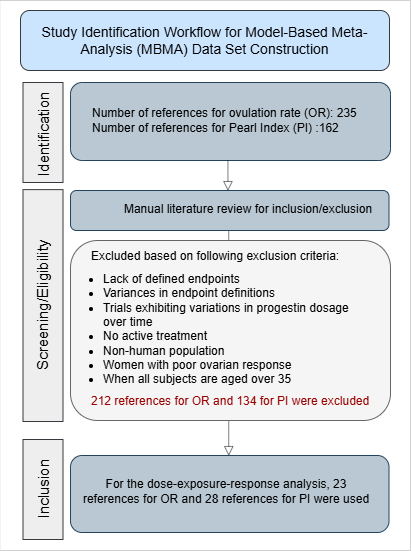


**Figure S1.** Study Identification workflow for Model-Based Meta-Analysis (MBMA) data set creation


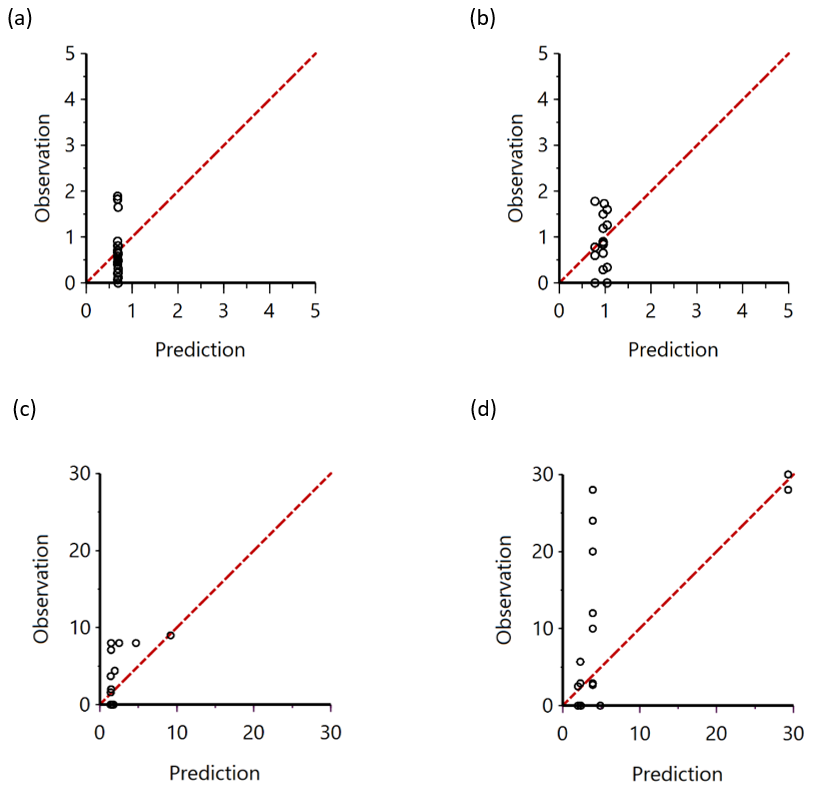


**Figure S2.** Goodness-of-Fit plot of final operational model of agonism model for Pearl index (PI) and ovulation rate (OR) in exposure-response analysis. Population predicted verses observed PI of (a) DRSP and (b) LNG, and population predicted verses observed OR of (c) DRSP and (d) LNG.


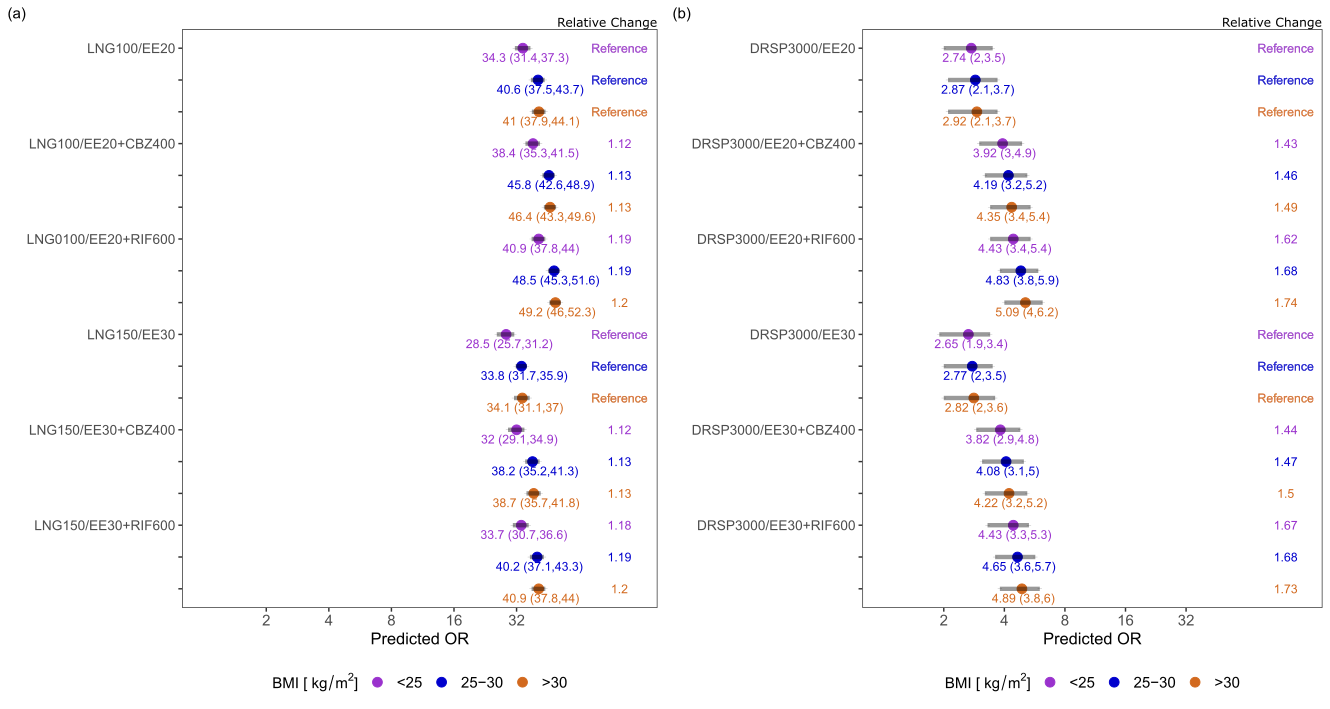


**Figure S3.** (a) Predicted ovulation rate (OR) with 95% CI of LNG150/EE30 (LNG 150 µg + EE 30 µg) and LNG100/EE20 (LNG 100 µg + EE 20 µg) co-administered with CYP3A4 inducers across BMI groups. Reference group includes women with no CYP3A4 induction. (b) Predicted OR with 95% CI of DRSP3000/EE20 (DRSP 3000 µg + EE 20 µg) and DRSP3/EE30 (DRSP 3000 µg + EE 30 µg) co-administered with CYP3A4 inducers across BMI groups. Reference group includes women with no CYP3A4 induction.

CBZ, carbamazepine; RIF, rifampicin; EE, ethinyl estradiol; LNG, levonorgestrel; DRSP, drospirenone.
